# Interleukin-2/Anti-Interleukin-2 Immune Complex Attenuates Cardiac Remodeling after Myocardial Infarction through Expansion of Regulatory T Cells

**DOI:** 10.1155/2016/8493767

**Published:** 2016-04-06

**Authors:** Zhipeng Zeng, Kunwu Yu, Long Chen, Weihua Li, Hong Xiao, Zhengrong Huang

**Affiliations:** ^1^Laboratory of Cardiovascular Immunology, Key Laboratory of Biological Targeted Therapy of the Ministry of Education, Institute of Cardiology, Union Hospital, Tongji Medical College of Huazhong University of Science and Technology, Wuhan 430000, China; ^2^Department of Cardiology, The First Affiliated Hospital of Xiamen University, Xiamen 361000, China; ^3^The Fourth Hospital of Wuhan, Wuhan 430000, China

## Abstract

CD4+CD25+Foxp3+ regulatory T cells (Treg cells) have protective effects in wound healing and adverse ventricular remodeling after myocardial infarction (MI). We hypothesize that the interleukin- (IL-) 2 complex comprising the recombinant mouse IL-2/anti-IL-2 mAb (JES6-1) attenuates cardiac remodeling after MI through the expansion of Treg. Mice were subjected to surgical left anterior descending coronary artery ligation and treated with either PBS or IL-2 complex. The IL-2 complex significantly attenuates ventricular remodeling, as demonstrated by reduced infarct size, improved left ventricular (LV) function, and attenuated cardiomyocyte apoptosis. The IL-2 complex increased the percentage of CD4+CD25+Foxp3+ Treg cells, which may be recruited to the infarcted heart, and decreased the frequencies of IFN-*γ*- and IL-17-producing CD4+ T helper (Th) cells among the CD4+Foxp3− T cells in the spleen. Furthermore, the IL-2 complex inhibited the gene expression of proinflammatory cytokines as well as macrophage infiltrates in the infarcted myocardium and induced the differentiation of macrophages from M1 to M2 phenotype in border zone of infarcted myocardium. Our studies indicate that the IL-2 complex may serve as a promising therapeutic approach to attenuate adverse remodeling after MI through expanding Treg cells specifically.

## 1. Introduction

Myocardial infarction (MI) is a leading cause of death worldwide and usually leads to the progression of congestive heart failure (CHF) after MI. Despite considerable advance in the treatment and management of MI, the morbidity and mortality from CHF are steadily increasing and remain a major public health challenge [1]. The extent of myocardial necrosis and ventricular remodeling after MI, including the deterioration of left ventricular morphology and function, contribute to ventricular arrhythmias, CHF, and subsequent cardiovascular mortality [2]. Therefore, new promising strategies, particularly low-cost therapies, to attenuate adverse cardiac remodeling are urgently needed in the treatment after MI.

CD4+CD25+Foxp3+ regulatory T cells (Treg cells), which are responsible for maintaining immune homeostasis and tolerance, improve wound healing and ameliorate cardiac remodeling by dampening inflammatory injury in MI [3–5]. The adoptive transfer of CD4+CD25+ Treg ameliorates MI-induced ventricular remodeling and improves cardiac function following ischemia by suppressing inflammatory injury and fibrosis, and their deletion deteriorates cardiac inflammation and dysfunction [4, 6]. However, this method is limited by the lack of sufficient purity and numbers of Treg preparations for the transfer. Furthermore, many clinical trials for type 1 diabetes mellitus, graft versus host disease, and refractory Crohn's disease involving Treg transfer fail to obtain long-term cell engraftment or substantial clinical benefits [7]. Thus, expansion of endogenous Treg cells may be an attractive approach. For example, Treg expansion with CD28 superagonists (CD28SA) improves wound healing and reduces the inflammatory damage in rodent MI models [3, 8]. Recently, Tabares et al. revealed that a low dose of CD28SA was effective in the treatment of autoimmune and inflammatory conditions without catastrophic effects in humans [9].

Interleukin- (IL-) 2 is a critical cytokine involved in the proliferation and activation of Treg. Recent studies have shown that the IL-2 immune complex, which comprises IL-2 and anti-IL-2 mAb (JES6-1), selectively expands Treg cells up to sixfold and effectively inhibits the development and progression of atherosclerosis in ApoE−/− mice [10, 11]. Furthermore, some studies reported that the IL-2 complex achieved substantial therapeutic benefits in treating acute renal ischemia-reperfusion injury, angiotensin II-induced aortic aneurysm, islet allografts, and inflammatory bowel disease through the expansion of Treg cells [11–14]. However, there has been no report on the salutary effect of the IL-2 complex on cardiac function after MI. Here, we investigate whether the IL-2 complex attenuates wound healing and structural remodeling after myocardial infarction by expanding Treg in murine models.

## 2. Methods and Materials

### 2.1. Animals and Experimental Protocol

All experiments were approved by the Animal Care and Use Committee of Huazhong University of Science and Technology. Age matched 8- to 10-week-old male C57BL/6 mice underwent left anterior descending coronary artery (LAD) ligation as previously described [15]. After surgery, the surviving animals were randomly allocated into the IL-2 complex- or phosphate buffer saline- (PBS-) treated groups. For the IL-2/JES6-1 complex-treated group, a recombinant mouse IL-2/anti-IL-2 mAb (JES6-1) complex (1 *μ*g IL-2 plus 5 *μ*g anti-IL-2 mAb) was administered intraperitoneally in mouse for three consecutive days according to a previous study that showed the optimal expansion of Treg cells* in vivo* [11], after which they received two injections during the second week. The Sham group was subjected to the same procedure without LAD ligation. The control group mice were treated with PBS. The IL-2 and anti-IL2 mAb (JES6-1) were purchased from BioLegend (San Diego, CA, USA). Wound healing and apoptosis were detected on day 5, and cardiac functions and ventricular remodeling were examined on day 14 after MI.

### 2.2. Echocardiography

Transthoracic echocardiography was performed 14 days after MI on a Vevo 1100 high-resolution microimaging system (VisualSonics, Canada) equipped with a 14 MHz linear transducer. Two-dimensional short-axis views of the left ventricle (LV) were obtained at the level of the papillary muscle. M-mode images were used to measure LV wall thickness, LV end-systolic diameter (LVESD), and end-diastolic diameter (LVEDD). The percentages of fractional shortening (FS) and ejection fraction (EF) were calculated from the M-mode recording as previously reported [16].

### 2.3. Histopathological Staining and Immunofluorescence Analysis

On day 5 or 14, the hearts were excised and weighed immediately. The ratio of the heart weight (HW) to the body weight (BW) (HW/BW) was calculated. Hearts were fixed in 10% buffered formalin, were embedded in paraffin, and were cut into 7 *μ*m thick sections using a cryostat (CM 3050S, Leica). Masson's trichrome staining was performed for the assessment of infarct size, which was calculated as the sum of the epicardial and endocardial infarct circumference divided by the total LV epicardial and endocardial circumferences. For immunohistochemical staining on day 5, the rat anti-mouse CD68 monoclonal antibody (Abcam, UK) and the Foxp3 monoclonal antibody (eBioscience, San Diego, CA, USA) were used to detect macrophages and Foxp3+ lymphocytes, respectively.

For immunofluorescence analysis, CD68+Arg-1+ double positive macrophages were stained with the rat anti-mouse CD68 monoclonal antibody (Abcam, UK) and rabbit anti-mouse Arg-1 antibody (Abcam, UK), followed by a FITC-labeled goat anti-rat IgG antibody (Abcam, UK) and a CY3-labeled goat anti-rabbit IgG antibody (Abbkine, Redlands, CA, USA), respectively. CD68+iNOS+ double positive macrophages were also stained with rat anti-mouse CD68 antibody (Abcam, UK) and the rabbit anti-mouse iNOS antibody (Abcam, UK) followed by a FITC-labeled goat anti-rat IgG antibody (Abbkine, Redlands, CA, USA) and a CY3-labeled goat anti-rabbit IgG anti-body (Abcam, UK), respectively. The number of double positive cells was calculated as cells per area with identical exposure settings at 400-fold magnification using the Image-Pro Plus 6.0 software (Media Cybernetics).

To detect apoptosis, TUNEL (terminal deoxynucleotidyl transferase-mediated dUTP nick end-labeling) staining was conducted on 4 *μ*m thick paraffin-embedded sections according to the manufacturer's instructions (Roche, Indianapolis, IN, USA). After labeling with TUNEL, sections were incubated with monoclonal anti-*α*-Actinin (sarcomeric) antibody (clone EA53; Sigma) followed by the CY3-labeled anti-mouse IgG antibody (Invitrogen, Waltham, MA, USA). DAPI staining was used to count the total number of nuclei. The apoptosis index was measured as the percentage of TUNEL-positive myocyte nuclei per the total number of nuclei.

### 2.4. Flow Cytometric Analysis

The detections of CD4+CD25+Foxp3+ Treg cells, CD4+IFN-*γ*+ T cells, and CD4+IL-17+ T cells in spleens or mediastinal lymph nodes (mLNs) was performed as previously described [17]. The lymphocytes from the spleen or LNs were isolated. For the analysis of Treg cells, cells were stained with anti-CD4-FITC mAb (eBioscience) and anti-CD25-APC mAb (eBioscience) and then stained with anti-Foxp3-PE mAb (eBioscience) after fixation and permeabilization according to the manufacturer's instructions. For detecting Th1 (CD4+IFN-*γ*+) and Th17 (CD4+IL-17+), cells were stimulated with 20 ng/mL PMA, 1 *μ*g/mL ionomycin, and 2 *μ*mol/mL monensin for 5 hours at 37°C in a 5% CO_2_ environment. Anti-CD4-FITC mAb staining was performed before fixation and permeabilization. Permeabilized cells were subsequently stained with anti-Foxp3-APC (eBioscience) and anti-IFN-*γ*-PE mAb (eBioscience) or anti-IL-17A-PE mAb (BD Biosciences). Isotype control antibodies were used to confirm antibody specificity and to enable correct compensation. Cells acquisition was performed using a flow cytometer (FACSCalibur, BD Biosciences), and all of the data was analyzed using FlowJo software (Tree Star Inc.).

### 2.5. Real-Time RT-PCR Analysis

Total RNA was extracted from the infarcted myocardium of 5-day-old hearts using the TRIzol reagent (Invitrogen, Waltham, MA, USA). Its concentration and purity were detected using a NanoDrop 2000 (Thermo Scientific, Waltham, MA, USA). cDNA was synthesized using the RNA PCR kit (Takara, Japan) and was used as a template. Quantitative real-time PCR was performed on an ABI Prism 7900 Sequence Detection System (Applied Biosystems, Waltham, MA, USA) using SYBR Green Master Mix (Takara, Japan). All results were normalized to *β*-actin and analyzed as previous described [18]. Sequences of primers are as follows: 5′-CCCGGCCTGGTCTGCTCCTC-3′ and 5′-GTGGCGGGGTGGTTTCTGAAGTAG-3′ for Foxp3; 5′-GCCCGAACCCCCATTGCTGTCC-3′ and 5′-AGGCGTATCAGTGGGGGTCAGCAGC-3′ for TGF-*β*; 5′-TCAAATCTCGCAGCAGCACATC-3′ and 5′-CGTCACACACCAGCAGGTTATC-3′ for IL-1*β*; 5′-GAAATGATGGATGCTACCAAACTG-3′ and 5′-GACTCTGGCTTTGTCTTTCTTGTT-3′ for IL-6; 5′-ACCCTCACACTCACAAACCA-3′ and 5′-ATAGCAAATCGGCTGACGGT-3′ for TNF-*α*; 5′-TACTTGGACGGATAGATGGAGG-3′ and 5′-CATAGAAAGGAATCCACGCAGT-3′ for CD206; 5′-ATCAACACTCCCCTGACAACCA-3′ and 5′-TTCCATCACCTTGCCAATCC-3′ for Arg1; 5′-AAGAAGCACGTCTGGTTTGGAG-3′ and 5′-GGTCCATGTAGGCTACGCTGTT-3′ for collagen I; 5′-GTGGCAATGTAAAGAAGTCTCTGAAG-3′ and 5′-GGGTGCGATATCTATGATGGGTAG-3′ for collagen III; 5′-CACTCCAATCGTCCCTAC-3′ and 5′-AGACTCACCGCTCTTCAT-3′ for OPN; 5′-GTGACGTTGACATCCGTAAAGA-3′ and 5′-GTAACAGTCCGCCTAGAAGCAC-3′ for *β*-actin.

### 2.6. Statistical Analysis

The data are presented as the mean ± SD and analysis was performed with SPSS 16.0. The comparisons between two groups were carried out using Student's *t*-test. One-way ANOVA was performed for multiple comparisons between three groups. A value of *P* < 0.05 was considered to be statistically significant.

## 3. Results

### 3.1. Expansion of the CD4+CD25+Foxp3+ Lymphocyte Treated with the IL-2 Complex

**Figure 1:**
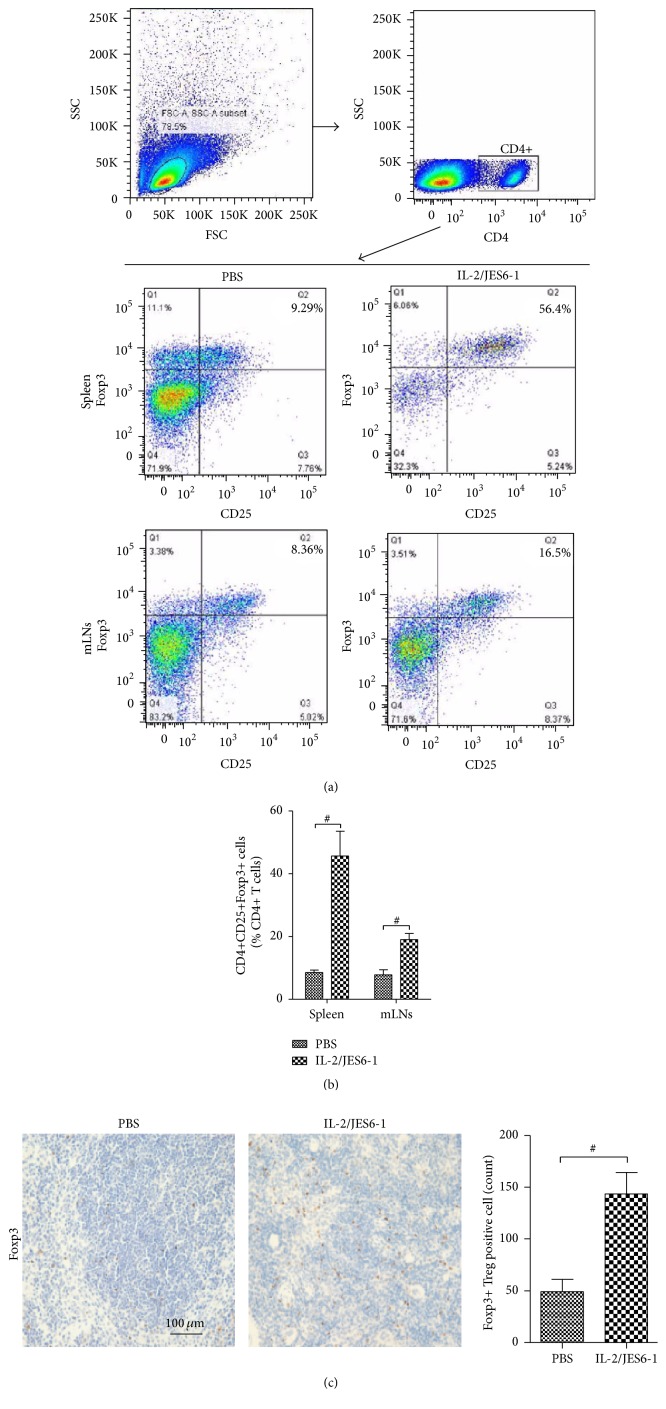
Expansion of CD4+CD25+Foxp3+ Tregs in both spleen and mediastinal lymph nodes (mLNs) after treatment with IL-2/JES6-1 complex* in vivo*. PBS or the IL-2/JES6-1 complex was administered intraperitoneally in mouse for three consecutive days. CD4+CD25+Foxp3+ Treg cells from splenocytes or mLNs cells were stained and analyzed by flow cytometry on day 5. (a) Representative results of the proportion of CD4+CD25+Foxp3+ Treg cells in the spleen and mLNs analyzed by flow cytometry. (b) The percentage of CD4+CD25+Foxp3+ Treg cells within the CD4+ T cell population was enhanced in both spleen and mLNs in the IL-2/JES6-1 group. (c) The numbers of Foxp3+ T cells in spleen were also increased in the IL-2/JES6-1 group by immunohistochemical staining (*n* = 6 per group). Original magnification ×200. ^#^ *P* < 0.05 versus the PBS group.

C57BL/6 mice were injected daily with the IL-2 complex (IL-2 : IL-2 mAb = 1 *μ*g : 5 *μ*g) or with PBS as a control for 3 consecutive days. Consistent with previous studies, the IL-2 complex markedly increased the percentage of CD4+CD25+Foxp3+ T cells in the CD4+ T cell population on day 5, with a 5.4-fold increase in the spleen (*P* < 0.05) and a 2.4-fold increase in mLNs (*P* < 0.05) (Figure 1(a)). The dramatic increase of Foxp3+ Treg cells in the spleen of mice injected with the IL-2 complex was also proved using immunohistochemical methods (Figure 1(c)).

### 3.2. The IL-2 Complex Reduces Infarct Size and Improves Cardiac Function

**Figure 2:**
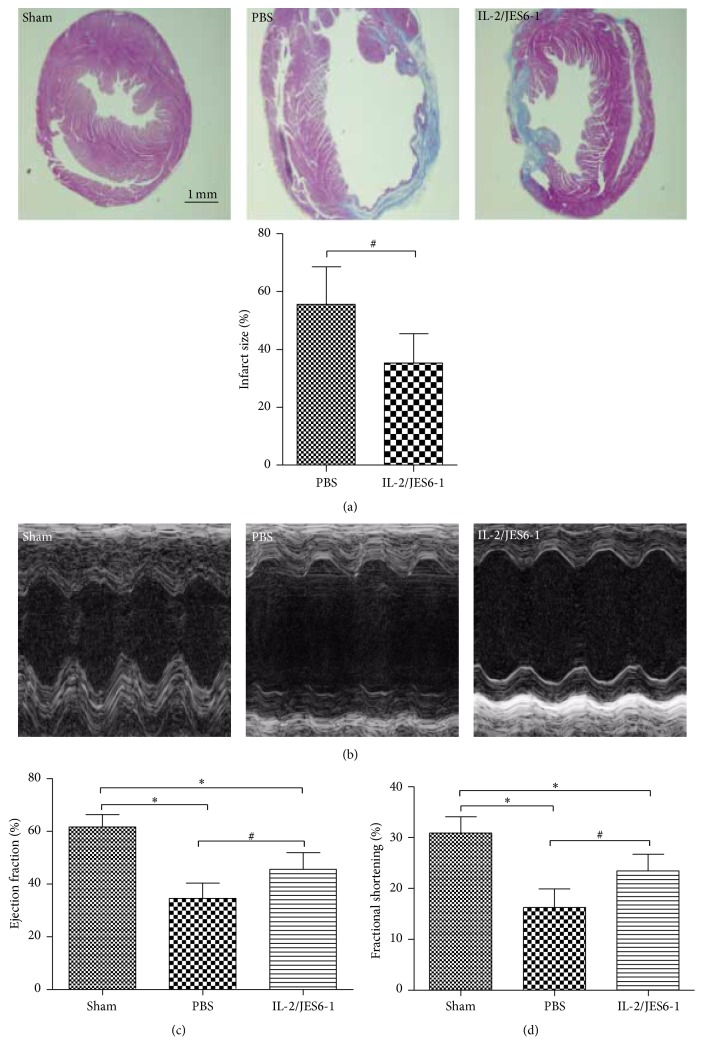
The IL-2/JES6-1 complex attenuates cardiac remodeling on day 14 after MI. (a) Trichrome stained heart sections showed that infarct size was reduced in the IL-2/JES6-1 complex group (*n* = 6 per group). (b) M-mode echocardiographic images of the left ventricle on day 14 after MI. Left ventricular ejection fraction (c) and fractional shortening (d) on day 14 after MI. ^*∗*^ *P* < 0.05 versus the Sham group; ^#^ *P* < 0.05 versus the PBS group.

To directly investigate the role of the IL-2 complex in ventricular remodeling after MI, we induced acute MI* via* permanent LAD ligation in mice. Mice were injected either with the IL-2 complex or with PBS for 3 consecutive days beginning on day 1 after surgery; therefore, they were injected twice the following week. The cardiac function of these mice was assessed by echocardiography on day 14 before the mice were euthanized. Although the survival was similar between the two groups, the IL-2 complex significantly reduced the infarct size (35.21 ± 10.22% versus 55.64 ± 12.85%; *P* < 0.05, Figure 2(a)). As shown in Table 1 and Figure 2, the values of the left ventricular ejection fraction (EF)% and ventricular fractional shortening (FS)% were markedly larger than control groups. The values of left ventricular end-systolic diameter (LVESD) and left ventricular end-diastolic diameter (LVEDD) in the IL-2 complex group were also significantly decreased compared to the values in the control groups. Moreover, the IL-2 complex also decreased heart weight/body weight ratios (*P* < 0.05 versus PBS group, Table 1). These results clearly indicated that the IL-2 complex significantly ameliorated ischemic injury and adverse structural ventricular remodeling after MI.

### 3.3. The IL-2 Complex Selectively Expands Treg Cells and Increases Recruitment of Treg in the Infarcted Heart

**Figure 3:**
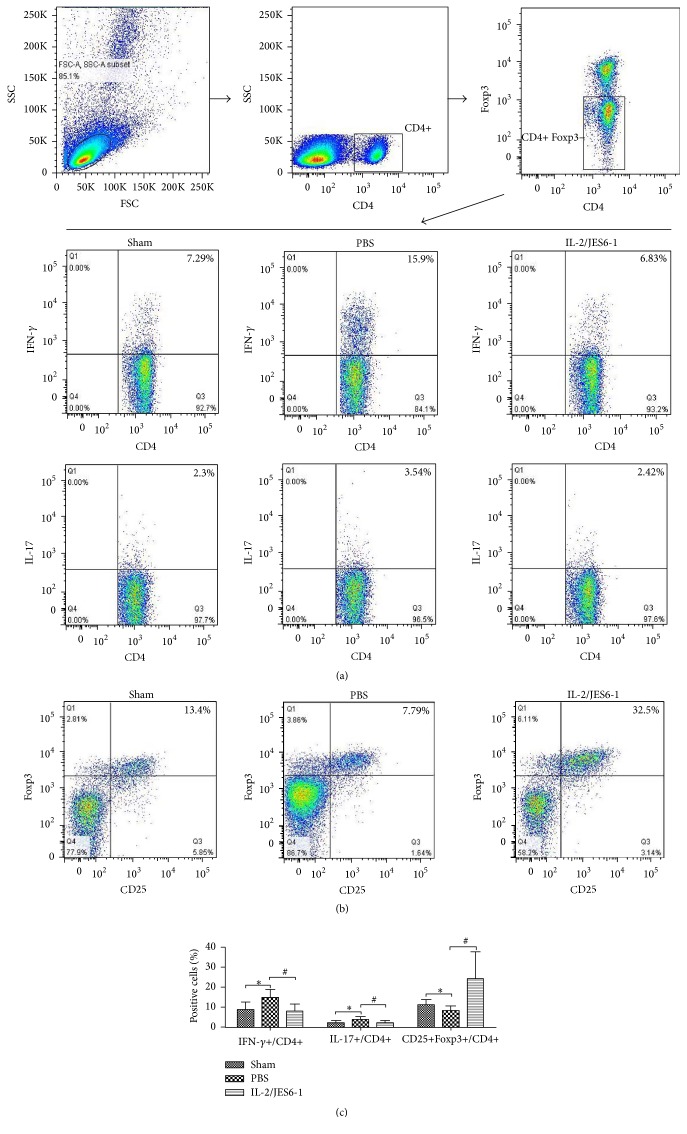
The IL-2/JES6-1 complex increases Treg cells and reduces Th1/17 cells. Splenocytes from 14 days after MI mice were stimulated with PMA/ionomycin/monensin, and CD4+IFN-*γ*+ (Th1) or CD4+IL-17+ (Th17) cells among CD4+ Foxp3− T cells were determined by intracellular staining for IFN-*γ*+ or IL-17, respectively, as described in Section 2. Quantification of percentage of CD4+IFN-*γ*+ cells and CD4+IL-17+ cells was analyzed by Flow Jo software. ^*∗*^ *P* < 0.05 versus the Sham group; ^#^ *P* < 0.05 versus the PBS group.

Previous studies have shown that Treg limits the differentiation of Th1 and Th17 lymphocytes, and both subsets play a pathogenic role in MI-induced adverse ventricular remodeling and acute coronary syndrome (ACS) [19, 20]. Because treatment with the IL-2 complex increased Treg cells in the spleen and mLNs, we further analyzed the effect of the IL-2 complex on differentiations of Th1 and Th17 subsets in the spleen. After 14 days of therapy with the IL-2 complex, Treg cells were selectively expanded by 2.8-fold in the spleen (CD4+CD25+Foxp3+ Treg cells: Sham, 11.29 ± 2.55%; PBS, 8.23 ± 2.31%; IL-2/JES6-1, 24.35 ± 13.36%; *P* < 0.05 versus PBS, Figure 3(c)). Furthermore, the populations of the IFN-*γ*-secreting Th1 subtype and IL-17-secreting Th17 subtype in CD4+Foxp3− T cells from the spleen were significantly reduced in groups treated with the IL-2 complex (IFN-*γ*+CD4+: Sham, 8.86 ± 3.47%; PBS, 14.94 ± 3.88%; IL-2/JES6-1, 8.01 ± 3.48%; IL-17+CD4+: Sham, 2.25 ± 1.07%; PBS, 3.86 ± 1.36%; IL-2/JES6-1, 2.2 ± 1.1%; *P* < 0.05 versus PBS; Figure 3). In addition, Foxp3+ cells were significantly increased by 4.3-fold (Figure 5(a)), and Foxp3 mRNA expression increased nearly by 3.4-fold (Figure 5(b)) in the infarct myocardium on day 5. Our results suggested that the amplified Treg cells in secondary lymphoid tissues may be recruited to the ischemic myocardium and may suppress the differentiation of CD4+ T cells toward the Th1 and Th17 subtypes.

### 3.4. The IL-2 Complex Attenuates MI-Induced Cardiomyocyte Apoptosis

**Figure 4:**
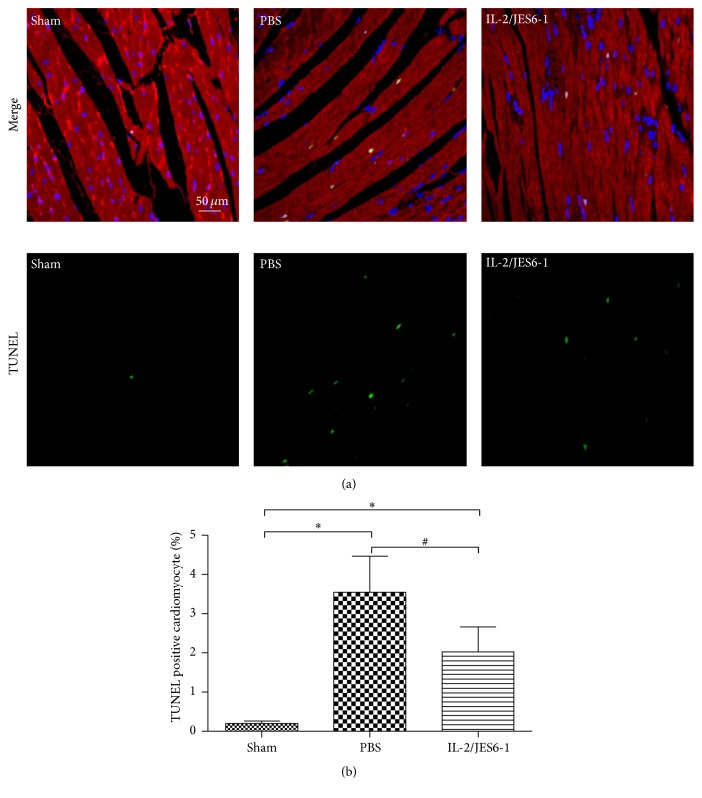
The IL-2/JES6-1 complex attenuates myocardial apoptosis after MI. (a) Representative images of TUNEL staining for myocardial apoptosis (green), DAPI (blue) for nuclear staining, and anti-*α*-Actinin (red) for cardiomyocytes in peri-infarct zone on day 5 after MI. Original magnification ×400. (b) Quantitative analysis of percentages of TUNEL-positive nuclei (*n* = 6 per group). ^*∗*^ *P* < 0.05 versus the Sham group, ^#^ *P* < 0.05 versus the PBS group.

The apoptosis of cardiomyocyte in the ischemic zone contributes to adverse ventricular remodeling, and previous studies have proven that the Treg cells attenuate cardiomyocyte apoptosis induced by MI. To detect the effect of the IL-2 complex on cardiomyocyte apoptosis, heart sections were stained with TUNEL on five days after MI. As shown in Figure 4, there was minimal detection of cardiomyocyte apoptosis in the Sham, but significant enhancement in the PBS group (percentage of apoptosis; Sham, 0.19 ± 0.06; PBS, 3.55 ± 0.92; *P* < 0.05 versus Sham). However, the IL-2 complex markedly reduced the apoptosis of cardiomyocytes in the border zone of the LV infarct (percentage of apoptosis: IL-2/JES6-1, 2.02 ± 0.63; *P* < 0.05 versus PBS; Figure 4). These results indicated that the IL-2 complex attenuates cardiomyocyte apoptosis after MI.

### 3.5. The IL-2 Complex Reduces the Infiltration of Macrophage to the Heart and Decreases the mRNA Expression of Proinflammatory Cytokine

**Figure 5:**
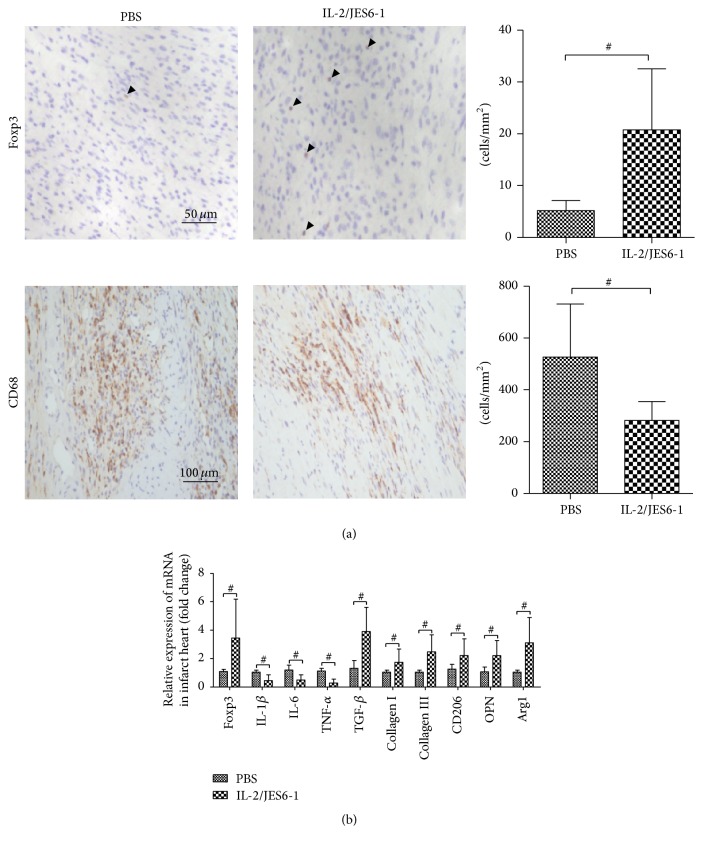
(a) The infiltration of Foxp3+ Treg cells and CD68+ macrophage in the infarcted heart was compared between the two groups (*n* = 6 per group) on day 5. Original magnification ×400 (upper), ×200 (down). (b) mRNA expression levels of cytokines and M1 and M2 macrophages markers in the infarcted heart at day 5. ^#^ *P* < 0.05 versus the PBS group.

To determine whether the protective role of the IL-2 complex in MI is mediated through the reduction of inflammatory injury, we investigated the infiltration of inflammatory cell and cytokine expression in the heart* via* immunohistochemistry and real-time PCR methods, respectively. Macrophages have been shown to be mobilized and recruited into the ischemic myocardium and are responsible for ventricular remodeling. The infiltration of macrophages was reduced by 46.6% in the group that was treated with the IL-2 complex (Figure 5(a)). The mRNAs levels of the proinflammatory cytokines TNF-*α*, IL-1*β*, and IL-6 were dramatically reduced in the IL-2 complex-treated compared to controls. In addition, Foxp3, TGF-*β*, collagen I, and collagen III were higher in the IL-2 complex-treated group than in the control group. Previous results suggested that IL-1*β* and TNF-*α* induced macrophage polarization toward M1 proinflammatory macrophages rather than M2 anti-inflammatory macrophages [21]. Consistent with this finding, we found that the IL-2 complex increased the mRNA synthesis of CD206, arginase 1 (Arg1), and osteopontin (OPN), whereas the levels of TNF-*α* were decreased (Figure 5(b)). These results suggest that the IL-2 complex may suppress the infiltration of macrophage in the infarcted heart and inhibit proinflammatory macrophage differentiation and activation in the infarcted myocardium.

### 3.6. The IL-2 Complex Downregulates the Number of M1 Macrophages but Upregulates the Number of M2 Macrophages in the Myocardium

**Figure 6:**
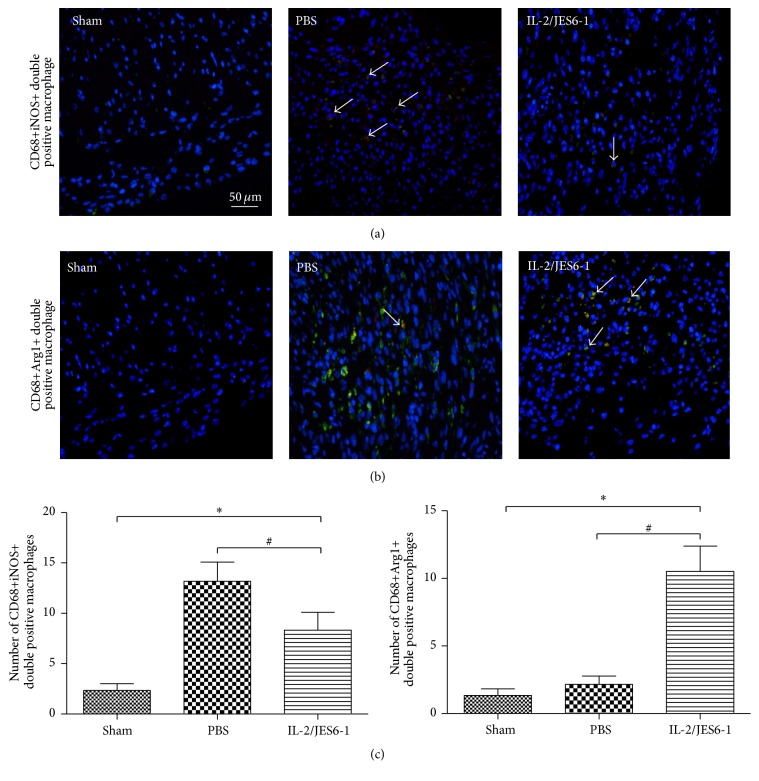
Immunofluorescent double staining of myocardial tissue with antibodies to CD68 and iNOS (a) or to CD68 and Arg-1 (b). Green indicates CD68, red iNOS or Arg-1, and blue DAPI-stained cellular nuclei. CD68+iNOS+ or CD68+Arg-1+ double positive macrophage shows yellow area (arrows). (c) The number of CD68+iNOS+ or CD68+Arg-1+ double positive macrophages in myocardial tissue from the Sham, PBS, and IL-2/JES6-1 treated mice (*n* = 7 per group). Original magnification ×400; ^*∗*^ *P* < 0.05 versus the Sham group, ^#^ *P* < 0.05 versus the PBS group.

To analyze the number of M1 and M2 macrophages in the myocardium, we used immunofluorescence to label CD68, which is a specific cell surface marker of macrophages. We also labeled iNOS and Arg-1, which are the specific markers of M1 or M2 macrophages, respectively. MI induced a striking infiltration of CD68+iNOS+ double positive macrophages compared with the Sham group. However, after treatment with the IL-2 complex, the number of CD68+iNOS+ double positive macrophages was markedly reduced (Figure 6). Conversely, the number of CD68+Arg-1+ double positive macrophages was minimal in the Sham and the PBS groups. In the IL-2 complex-treated group, the number of CD68+Arg-1+ double positive macrophages was significantly upregulated, although the total number of CD68+ macrophages was reduced (Figure 6). Taken together, these data indicate that the IL-2 complex not only inhibits the infiltration of macrophage, but also induces M2 macrophages differentiation in the myocardium of MI mice.

## 4. Discussion

In the current study, our results demonstrate that the IL-2 complex significantly inhibits an inflammatory response and attenuates ventricular remodeling after MI. The underlying mechanisms are mainly associated with the selective expansion of Treg cells and the suppression of proinflammatory Th1 and Th17 subtypes, leading to the shift of macrophages from a M1 to M2 phenotype in the myocardium.

IL-2 is a monomeric secreted cytokine that plays a vital role in the homeostasis, activity, and maintenance of Treg cells in the periphery through the IL-2 receptor complex (IL-2R*α*, IL-2R*β*, and IL-2R*γ*) [22]. Two recent clinical studies showed that low-dose IL-2 administration had a therapeutic benefit in alleviating chronic graft versus host disease (GVHD) and chronic hepatitis C virus- (HCV-) mediated vasculitis by augmenting Treg cell counts and function without activating T effector cells [23, 24]. However, NK cells were also increased when they were treated with low-dose IL-2. Boyman et al. demonstrated that the IL-2 complex formed by IL-2 and anti-mouse IL-2, JES6-1, preferentially expanded Treg cells with little or no effect on other cells [25]. Subsequently, Treg cell expansion induced by the administration of the IL-2 complex has therapeutic applications in graft injection and autoimmune and inflammatory diseases without inducing toxicity [11]. The interaction of the IL-2 complex with Treg cell is mainly dependent on JES6-1, which blocks the IL-2R*β* binding site on mIL-2. The antibody (JES6-1) helps IL-2 to target high-affinity interactions with IL-2R*α* (CD25), which is highly expressed on the surface of Treg cells [14, 22]. A previous study had found that the IL-2 complex with 5 *μ*g of mAb and 1 *μ*g of IL-2 led to near optimal expansion of Treg cells in mice [11]. Thus, we chose this protocol for subsequent experiments. Consistent with previous studies, we found that the IL-2 complex effectively amplified Treg population in the spleen and exerted a protective effect on adverse remodeling after MI.

Treg cells, which are responsible for maintaining immune homeostasis and tolerance, play a critical role in wound healing and the resolution of the inflammatory response after MI or ischemic/reperfusion injury [3, 5]. Recent studies have suggested that either adoptive transfer of Tregs or the endogenous expansion by agonists has beneficial effects on hypertension, atherosclerosis, fibrosis, and MI [6, 10, 26]. On the other hand, Treg ablation by anti-CD25 or by genetic method exacerbates cardiac inflammation and impairs wound healing after MI [3]. Treg cells may attenuate the inflammatory response and adverse remodeling and protect against apoptosis after MI. A recent study demonstrated that Treg cells exert their beneficial effects by limiting the infiltration of proinflammatory myeloid cells and facilitating the differentiation of M2 macrophages [3]. Two of the most promising approaches to expanding endogenous Treg cells in animal models involve either CD28-superagonistic monoclonal antibodies or IL-2/anti-IL-2 complexes [27]. Although superagonistic anti-CD28 treatment reduces cardiac or brain damage after ischemic injury in mice through amplification of Treg cells, it has failed in clinical trial as the treatment leads to catastrophic effects involving a massive cytokine storm and multiorgan failure [27]. However, a recent phase I trial revealed that a low dose of CD28SA had little catastrophic effects in humans [9]. Alternatively, the IL-2/anti-IL-2 complex may offer a promising approach for Treg therapy. The recruitment of Treg cells in the border zone of the infarcted myocardium, where they may contribute to protecting against cardiomyocyte apoptosis and inhibiting inflammation-induced injury, is enhanced by the IL-2 complex. Our results provide an alternative strategy that surmounts the obstacles of adoptive transfer and catastrophic damage caused by CD28-superagonistic antibodies.

Monocytes and macrophages play a critical role in protecting against adverse ventricular remodeling after experimental infarction [28]. Generally, M1 macrophages, which produce proinflammatory cytokines such as TNF-*α*, IL-1*β*, and IL-6, are associated with myocardial damage and the maladaptive repair response after MI. In addition, M2 macrophages, which secrete anti-inflammatory cytokines and growth factors such as IL-10, TGF-*β*, and VEGF, are responsible for resolving the inflammation and promoting wound healing and angiogenesis. Although the two monocyte subsets display different phases and functions after MI, monocytes have high plasticity and develop into either proinflammatory or anti-inflammatory macrophages that mainly depend on the microenvironment of the infarcted myocardium. Thus, reprograming the polarity of monocytes and macrophages is a promising therapeutic approach in reducing the inflammatory response and improving wound healing after MI. A recent study revealed that silencing of the transcription factor known as the interferon regulatory factor 5 (IRF5), which serves as a critical regulator of macrophage polarization, accelerated inflammation resolution in healing infarcts by modulating the macrophage phenotype and improved post-MI ventricular remodeling and CHF [29].

A previous study revealed that fostering the differentiation of M2 macrophages could improve wound healing and inflammatory resolution after MI through the expansion of Treg cells induced by anti-CD28 or other methods [30]. TNF-*α*, IFN-*γ*, and other inflammatory cytokines are responsible for the polarization of M1 macrophages. Our results found that the increased synthesis of TGF-*β* and the reduced expression of TNF-*α* and IL-6 could foster the differentiation of M2 macrophages, which was verified by the significant increase in the expression of Arg-1, CD206, collagen I, and collagen III in the infarct heart. In addition, the number of the infiltrated CD68+ macrophages was significantly decreased in the border zone of the infarcted heart in the IL-2 complex group. This process mainly contributed to the reduced synthesis of TNF-*α*, IL-1*β*, and IL-6, which led to M1 macrophage polarization. Similarly, immunofluorescence staining further demonstrated that the IL-2 complex downregulated the number of M1 macrophages but upregulated the number of M2 macrophages in the myocardium. Collectively, Treg expansion by the IL-2 complex treatment fosters M2 macrophage differentiation, which improves wound healing. However, the exact origin of M2 macrophages remains be determined and needs further investigation.

The activation of CD4+ T cells facilitates wound healing and survival after MI, and the protective role of CD4+ T cells may involve a key CD4+ T cell subset known as regulatory T cells [15]. However, the adoptive transfer of CD4+ T cells derived from wild type mice but not IFN-*γ* knockout mice into the RAG-deficient mice has adverse influence on would healing and increases the infarct size [31]. These results suggest that IFN-*γ*+CD4+ T cells have a negative effect on wound healing. Moreover, IL-17A plays a pathogenic role in ventricular remodeling by inducing the inflammatory response and enhancing apoptosis after MI [32, 33]. IL-17A in the myocardium is mainly derived from *γδ* T cells rather than the Th17 polarization of CD4+ T cells [32]. The effect of the IL-2 complex on *γδ* T cells requires further investigation. Treg cells expanded with the IL-2 complex might suppress the differentiation of Th1 and Th17 in secondary lymphoid tissues. Furthermore, the differentiation and activation of proinflammatory M1 macrophages were also reduced in the infarct myocardium. These effects contribute to the resolution of the inflammatory response and wound healing after MI.

In conclusion, our data further emphasize the concept that regulatory T cells play a protective role in MI-induced ventricular remodeling. The IL-2 complex exerts protective effects by directly inhibiting the infiltration of inflammatory macrophages and by facilitating the polarization of anti-inflammatory M2 macrophages, which attenuate cardiomyocyte apoptosis and the local inflammatory responses. The expansion of Treg cells by the IL-2 complex may be a potentially valuable approach to improving ischemic heart disease.

## Figures and Tables

**Table 1:** Characteristics of mice at 2 weeks after Sham or MI operation.

Parameters	Sham (*n* = 10)	Fourteen days after MI	
		PBS (*n* = 11)	IL-2/JES6-1 (*n* = 10)
Body weight (BW, g)	25.42 ± 1.61	24.78 ± 2.02	24.86 ± 1.49
Heart weight (HW, mg)	123 ± 7.68	158.15 ± 5.84^*∗*^	145.23 ± 4.24^*∗*#^
HW/BW (mg/g)	4.83 ± 0.38	6.38 ± 0.36^*∗*^	5.73 ± 0.57^*∗*#^
LVEDD, mm	4.03 ± 0.32	5.21 ± 0.40^*∗*^	4.34 ± 0.44^*∗*#^
LVESD, mm	3.12 ± 0.28	4.15 ± 0.50^*∗*^	3.42 ± 0.41^*∗*#^
EF, %	61.61 ± 6.70	34.58 ± 6.60^*∗*^	45.53 ± 6.70^*∗*#^
FS, %	30.92 ± 3.61	16.27 ± 4.01^*∗*^	23.41 ± 5.28^*∗*#^
Heart rate, bpm	479 ± 30.77	489 ± 34.19	476 ± 44.99

Values are means ± SD. bpm indicates beats per minute; LVEDD: LV end-diastolic diameter; LVESD: LV end-systolic diameter; EF: ejection fraction; FS: fractional shortening. ^*∗*^ *P* < 0.05 versus the Sham group, ^#^ *P* < 0.05 versus the PBS group.
